# Internalizing symptoms in adolescence are modestly affected by symptoms of anxiety, depression, and neurodevelopmental disorders in childhood

**DOI:** 10.1186/s12888-022-03875-6

**Published:** 2022-04-01

**Authors:** Sabrina Doering, Henrik Larsson, Linda Halldner, Christopher Gillberg, Ralf Kuja-Halkola, Sebastian Lundström

**Affiliations:** 1 Centre for Ethics, Law and Mental Health (CELAM), Institute of Neuroscience and Physiology, Sahlgrenska Academy, University of Gothenburg, Gothenburg, Sweden; 2 Department of Medical Epidemiology and Biostatistics, Karolinska Institutet, Stockholm, Sweden; 3 Department of Clinical Sciences, Umeå University, Umeå, Sweden; 4 Gillberg Neuropsychiatry Centre, Institute of Neuroscience and Physiology, Sahlgrenska Academy, University of Gothenburg, Gothenburg, Sweden

**Keywords:** Anxiety, Depression, Internalizing disorders, Neurodevelopmental disorders, Developmental psychopathology, Child, Adolescent

## Abstract

**Background:**

Internalizing disorders, such as anxiety and depressive disorders, are common mental disorders in young people, but a detailed understanding of the symptom continuity from childhood to adolescence that additionally includes a variety of neurodevelopmental disorder (NDD) symptoms is lacking. We therefore aimed to assess the extent to which parent-reported anxiety, depression, and NDD symptoms in childhood predict parent-reported internalizing symptoms in adolescence.

**Methods:**

We used the nation-wide population-based Child and Adolescent Twin Study in Sweden, comprising 4492 twins born in Sweden between 1998 and 2003 that were assessed at age 9, and then again at age 15. Linear regression in a structural equation modelling framework was used to analyze the data.

**Results:**

Overall, our results indicate that 15.9% of the variance in internalizing symptoms at age 15 can be predicted by anxiety, depression, and NDD symptoms at age 9. Anxiety and NDD symptoms in childhood predicted the largest amount of internalizing symptoms in adolescence.

**Conclusions:**

Adolescent internalizing symptoms are modestly affected by childhood symptoms of anxiety, depression, and NDDs, suggesting that they may represent different constructs across age. Future studies should further empirically investigate differences in etiology and trajectories of childhood versus adolescent internalizing symptoms.

**Supplementary Information:**

The online version contains supplementary material available at 10.1186/s12888-022-03875-6.

## Background

Internalizing disorders, such as anxiety and depressive disorders, are common mental disorders in children and adolescents and constitute an increasing burden by means of impairment in daily life [1–3]. Anxiety disorders affect between 9 and 32% of children and adolescents aged 3 to 18 years, while the rate of youth depressive disorders is estimated between 3 and 14% [4, 5].

While internalizing disorders and symptoms have been reported to increase in frequency from childhood to adolescence [6, 7], studies have identified different trajectories over time. For example, trajectories from childhood to adolescent anxiety and depression have been reported to be of homotypic [8, 9] and heterotypic nature [9]. Homotypic continuity refers to the continuity of the same construct over time (e.g., childhood anxiety predicts adolescent anxiety). Heterotypic continuity refers to a construct developing into another construct over time (e.g., childhood anxiety predicts adolescent depression). Considering that anxiety and depression symptoms have both distinct features (e.g., physiological hyperarousal in anxiety, low positive affect in depression) and common features such as negative affect [10], both homotypic and heterotypic trajectories are likely to occur. In an effort to examine the clustering of anxiety and depression symptoms in a community sample of children and adolescents, Cannon and Weems [11] reported a group with high levels of anxiety symptoms in 6 to 11-year-old children but with low levels of depression symptoms. However, the opposite was the case in 12 to 17-year-old adolescents, suggesting that internalizing symptoms may present themselves differently across age. In support of this notion, twin studies describe an increase of anxiety and depression symptoms during adolescence as well as high symptomatic stability in this age period, opposed to only moderate symptomatic stability in childhood. This change is also mirrored by differences in etiology: the heritability of anxiety and depression symptoms was found to be highest in childhood and then declined from adolescence and onwards [12].

Neurodevelopmental disorders (NDDs, e.g., attention-deficit/hyperactivity disorder (ADHD), autism spectrum disorder (ASD), developmental coordination disorder, tic disorder, and learning disorder) have their onset in the developmental period [13]. This group of disorders is believed to be present from birth, or at least at a very young age (with the exception of tic disorder), typically precedes the onset of internalizing disorders [14, 15], have been found to share etiology with internalizing disorders [16], and can be partly expressed through symptoms shared with anxiety and depressive disorders in children (e.g. [17–20]). For example, ADHD may be expressed through excessive crying, experiencing strong distress when separated from a caregiver, or disturbance of sleep [17, 18]. Children with ASD exhibit impairments in socio-communication manifested by avoidance or reluctance to social situations as well as restricted speech [17, 19, 20]. Taken together, it is possible that internalizing symptoms in childhood may be a partly distinct phenotype from internalizing symptoms in adolescence, with childhood internalizing symptoms potentially being more closely related to NDDs than adolescent internalizing symptoms. Today, there is a paucity of studies that have examined underlying differences in childhood and adolescent internalizing symptoms while simultaneously including a variety of NDDs.

The aim of the present study was to gain insight into the relationship between childhood and adolescent internalizing symptoms by analyzing data from 4,492 Swedish twins aged 9 and 15 years. Specifically, the aim was to assess the extent to which parent-reported symptoms of anxiety, depression, and NDDs in childhood predict parent-reported internalizing symptoms in adolescence.

## Methods

### Participants

The study is based on data from the Child and Adolescent Twin Study in Sweden (CATSS). CATSS is an ongoing, longitudinal, population-based study on somatic and mental health problems in children and adolescents (for details, see Anckarsäter et al. [21]). In short, since 2004, parents of all Swedish twins born from July 1992 and onwards are contacted in connection with the twins’ 9^th^ or 12^th^ birthday (CATSS-9/12, note that the first three years of the study also included 12-year-olds, however, these are not included in the present study) and asked to participate in a telephone interview, which includes measures of NDDs and mood disorder symptoms. The relevant measures for anxiety and depression at age 9 were included in CATSS from the 1998 cohort and onwards. When the twins reach the age of 15, families are contacted again and asked to fill out a web-based questionnaire, targeting various mental health problems (CATSS-15). CATSS-15 includes twins born from the 1^st^ of January 1994 and onwards. The overall response rate in CATSS-9/12 is approximately 70%. The overall response in CATSS-15 is 50% (parent report). In the present study, we included 12,644 individuals born between 1998 and 2003, out of which 11,437 had complete data on the NDD symptoms measure, 10,429 had complete data on the depression measure, and 9,016 individuals had complete data on the anxiety measure. A total of 8,949 individuals had complete data on all three measures at age 9 (i.e., no missing values on any of the three measures). Out of these 8,949 individuals, 4,492 individuals had complete data on all measures at both age 9 and age 15, which was the final number of individuals in the study sample. All measures (i.e., at age 9 and 15) were parent-reported.

## Measures at age 9

### Short Mood and Feelings Questionnaire (SMFQ)

The Short Mood and Feelings Questionnaire (SMFQ [22]) is a 13-item subscale from the longer 33-item Mood and Feelings Questionnaire, measuring core depressive symptoms in children and adolescents aged 6 – 17 years old. We used the parent report version. Parents are asked about their child’s feelings and actions over the timeframe of the previous two weeks. Items are scored as 0 (no), 1 (yes, to a certain degree) and 2 (yes). Total SMFQ scores range between 0 and 26. A total score of 12 or higher may indicate depression [23]. The SMFQ has been found to distinguish well between individuals with clinical depression and non-depressed individuals in a community sample [24] and has shown to correctly identify episodes of depression in youth [25]. In our sample, the scale has shown good internal consistency (Cronbach’s α = 0.83).

### Screen for Child Anxiety Related Disorders (SCARED)

The Screen for Child Anxiety Related Disorders (SCARED [26]) is a 41-item inventory designed to measure five child and adolescent anxiety symptom dimensions (i.e., panic disorder, generalized anxiety disorder, separation anxiety disorder, social phobia, and school anxiety/refusal). Items are scored as 0 (no), 1 (yes, to a certain degree) or 2 (yes), yielding a total score between 0 and 82. A total score greater than, or equal to, 25 may indicate an anxiety disorder [27]. The parent-reported version was used in this study. The SCARED was found reliable in terms of internal consistency and test–retest reliability, shows significant correlations with other measures for childhood anxiety disorders, and has been found to have good discriminant validity between anxiety and other mental disorders, as well as within anxiety disorders [26–29]. In our sample, the scale has shown good internal consistency (Cronbach’s α = 0.88).

### The Autism-Tics, ADHD and other Comorbidities inventory (A-TAC)

The Autism-Tics, ADHD and other Comorbidities inventory (A-TAC [30, 31]) is a fully structured 96-item parent-report telephone interview designed for large-scale epidemiological purposes. The A-TAC is based on symptom criteria and common clinical features of child and adolescent psychiatric disorders, including NDDs, as well as common other problem areas in child and adolescent psychiatry. Items are scored as 0 (no), 0.5 (yes, to some extent), and 1 (yes), and are divided into modules corresponding to diagnostic domains (e.g., Concentration & attention, Impulsiveness & activity, Social interaction, Language). Distributions, Cronbach’s α and heritability estimates are reported elsewhere [21]. Cross-sectional and longitudinal validation studies show good to excellent cross-sectional and predictive validity for, amongst others, ADHD, ASD, developmental motor coordination disorder, tic disorder, and learning disorder [31, 32]. A study by Pettersson et al. [33] described a broad general NDD factor accounting for a large proportion of the covariation among neurodevelopmental problems. To cover NDD symptoms, including their extensive phenotypic overlap both above and below diagnostic thresholds, a factor analysis of the 53 items on the A-TAC targeting ADHD, ASD, developmental coordination disorder, tic disorder and learning disorder was performed to examine their latent structure. Next, we fitted a one-factor model and created a single factor score for all individuals. To increase power, the NDD factor was created on all individuals (i.e., birth years 1992 and onwards), whereas statistical analyses are based on birth years 1998 and onwards (for descriptive statistics, see Table 1).

**Table 1:** Descriptive statistics for sample variables and scales

			**Scale range (Maximum score)**	**Mean (*SD*)**	***N***
NDD factor			0 – 23.45 (–^a^)	1.86 (2.84)	5708
Depression symptoms at age 9 (SMFQ)			0 – 25 (26)	0.82 (2.03)	5137
Anxiety symptoms at age 9 (SCARED)			0 – 63 (82)	4.72 (5.84)	4517
Internalizing symptoms at age 15 (SDQ)			0 – 10 (10)	1.27 (1.72)	4492
**Sex, *n* (%)**					
♂	♀				
2128 (47.4)	2364 (52.6)				
**Birth year *n* (%)**					
**1998**	**1999**	**2000**	**2001**	**2002**	**2003**
483 (10.8)	864 (19.2)	802 (17.9)	783 (17.4)	759 (16.9)	801 (17.8)

^a^Since the NDD factor is based on an individually calculated factor score, no meaningful maximum score is available

## Measures at age 15

### Strengths and Difficulties Questionnaire (SDQ)

CATSS-15 contains the Strengths and Difficulties Questionnaire (SDQ [34]). The SDQ is a brief, 25-item behavioral screening questionnaire for children and adolescents between 3 and 16 years and was developed to assess externalizing and internalizing symptoms. It consists of five subscales (i.e., conduct problems, hyperactivity, emotional symptoms, peer problems, and prosocial behavior) with five items in each subscale. In the present study, the emotional problems scale (parent report) was used as an indicator of internalizing symptoms in adolescence. The scale has 5 items with 3 response options: 0 (not true), 1 (somewhat true), and 2 (certainly true), yielding a total score between 0 and 10. The SDQ is widely used to screen for internalizing symptoms both in community and clinical settings [35, 36]. Studies of the Swedish version of the SDQ symptom scales have confirmed the factor structure of the original English SDQ [37]. Results from a validation study of the Swedish SDQ revealed that the parent version discriminates well between psychiatric and non-psychiatric individuals [38]. In our sample, the SDQ emotional symptoms scale has shown acceptable internal consistency (Cronbach’s α = 0.71).

### Statistical analyses

To examine the relationship between anxiety, depression, and NDD symptoms in childhood (i.e., at age 9), and internalizing symptoms in adolescence (i.e., at age 15), Cholesky decomposition models were employed. The model is comparable to a linear regression model within a structural equation modelling framework with regards to explained variance and covariance between the predictors and outcomes, but with the additional benefit that explained variance in the outcome can be partitioned into unique and shared contributions from the predictors. Thus, we aimed to estimate unique and shared explained variance of the three predictors (anxiety, depression, and NDD symptoms in childhood) on a fourth outcome variable (internalizing symptoms in adolescence). Next, we portioned the explained variance in the outcome into a part that was uniquely explained by each included predictor, and a part that was explained by the predictors but cannot be uniquely attributed to any one of the predictors – we refer to this part as ‘shared’. Each model has a focus on how much variance in the outcome (i.e., adolescent internalizing symptoms) is explained by the various predictors (i.e., anxiety, depression, and NDD symptoms in childhood). In total, we fitted four models to examine unique associations, including different combinations of childhood predictors: (1) anxiety symptoms, (2) anxiety and NDD symptoms, (3) anxiety and depression symptoms, and (4) all predictors. Since dependencies between twins can affect the precision of estimates (i.e., small standard errors and tight confidence intervals), we furthermore included an unstructured covariance matrix between twins in pairs for all variables (different covariance matrices for monozygotic and dizygotic twin pairs). All analyses were controlled for sex and birth year and were conducted in R 4.0.2 [39] using the R package ‘OpenMx’ [40].

## Results

Table 1 shows descriptive statistics for the anxiety, depression, and NDD symptom measures in childhood, and the adolescent internalizing symptoms measure. Overall, adolescent internalizing symptoms were neither explained by anxiety, depression, nor NDD symptoms to a substantial degree (Table 2). Childhood anxiety symptoms, and childhood anxiety and depression symptoms, respectively, explained approximately 12% of the variance in adolescent internalizing symptoms, with childhood depression symptoms having little unique effect. Childhood anxiety and NDD symptoms, and all childhood symptoms, respectively explained approximately 16% of the variance in adolescent internalizing symptoms. In the latter model, childhood anxiety symptoms explained 5.4% (95% CI 4.1% – 6.6%), and NDD symptoms explained 1.9% (95% CI 1.3% – 2.5%) of the variance in adolescent internalizing symptoms, while childhood depression symptoms did not uniquely explain any variance. Eight-point-six percent (95% CI 7.4% – 9.8%) of the explained variance in adolescent internalizing symptoms was shared between the childhood predictors.

**Table 2:** Unique and shared explained variance in adolescent internalizing symptoms by childhood anxiety, depression, and NDD symptoms

	**Models** **Percentage explained variance (95% Confidence Interval)**			
**Predictor**	Anxiety symptoms	Anxiety + NDD symptoms	Anxiety + depression symptoms	Anxiety + NDD + depression symptoms
NDD factor	–	2.2 (1.6–2.9)	–	1.9 (1.3–2.5)
Depression symptoms	–	–	0.3 (0.0–0.6)	0.0 (0.0–0.0)
Anxiety symptoms	12.2 (10.3–14.2)	6.7 (5.3–8.1)	7.4 (5.8–8.9)	5.4 (4.1–6.6)
Shared^a^	–	6.9 (6.1–7.7)	5.0 (3.9–6.0)	8.6 (7.4–9.8)
Total variance explained	12.2	15.8	12.7	15.9

All analyses were adjusted for sex and birth year

^a^‘Shared’ refers to the part that is explained by the predictors but cannot be uniquely attributed to any of the predictors in the models

## Discussion

The present study examined the relationship between symptoms of anxiety, depression, and NDDs in childhood and internalizing symptoms in adolescence in a longitudinal, population-based study of 4,492 Swedish twins. Overall, our results indicate that only a small amount of internalizing symptoms at age 15 can be explained by symptoms of anxiety, depression, and NDDs at age 9.

In line with previously reported patterns of continuity [8, 9] and findings from twin studies [12], we found that internalizing symptoms in adolescence can be partially demarcated from symptoms of anxiety and depression in childhood. In our sample, anxiety symptoms at age 9 predicted most of the variance while depression symptoms at age 9 predicted virtually none of the variance in internalizing symptoms at age 15, further supporting the idea that the expression of internalizing symptoms may be age-dependent [11]. That is, anxiety may be more dominantly expressed in childhood internalizing symptoms, while depression may be more characteristic of adolescent internalizing symptoms. It has been suggested that childhood depression is often overlooked, as its clinical presentation in children differs from that of adults [41]. Symptoms are expressed with less verbality and may resemble those of anxiety and several NDDs (e.g., crying, irritability, somatic complaints, behavioral problems). Studies have indicated that the trajectories of depression are dependent on whether symptoms first manifest in childhood or adolescence. For example, Rice et al. [42] compared one group whose depression symptoms emerged in early adolescence with a group whose symptoms emerged later in adolescence. The early-onset group was associated with an elevated genetic risk for childhood ADHD and other neurodevelopmental symptoms. Similarly, externalizing symptoms in childhood, but not in early adolescence, were found to be predictive of depression symptoms in adolescence [43], suggesting that childhood/early-onset forms of depression may be more closely associated with neurodevelopmental symptomatology. In the present study, childhood NDD symptoms explained negligible variance in adolescent internalizing symptoms, and although NDDs are common in both children and adolescents with anxiety disorders [44–47], it has been suggested that they may be more common in childhood anxiety disorders than in adolescent anxiety disorders [48]. Future studies have yet to investigate if different constellations of NDDs can be discerned in individuals with anxiety and depression in childhood vis-à-vis adolescence.

From an intervention or prediction perspective, our findings highlight the need to take age into account. It is possible that anxiety and depression in childhood require different intervention approaches than adolescent anxiety and depression. For example, Creswell et al. [49] note in a recent review on the treatment of anxiety disorders in children and adolescents that there is surprisingly little known about age-specific interventions of anxiety disorders, even though clinical characteristics for children and adolescents vary. The authors point out, however, that a major difference in the treatment of child versus adolescent anxiety lies in a greater focus on parental involvement in children. For depression, research generally shows that serotonin reuptake inhibitors appear to be less effective in young people than in adults when treating depressive disorders [50], while for children and adolescents with anxiety disorders, these medications generally work well in reducing the severity of anxiety [51], even more so in combination with cognitive behavioral therapy [52]. Although treating NDDs and childhood anxiety/depression indeed have positive effects on the child’s functioning, our results suggest that at least a large share of adolescent internalizing symptoms may be explained by factors unrelated to NDDs, anxiety and/or depression in childhood; likely due to an influx of genetic innovation in these age groups [12]. Nonetheless, childhood anxiety symptoms and NDD symptoms together explained roughly 16% of the variance in adolescent internalizing symptoms. Interventions towards childhood NDD symptoms and/or anxiety symptoms probably act in an interactive – rather than additive – fashion, which is why treatment of NDDs and/or anxiety symptoms may affect the development of adolescent internalizing symptoms.

The present study has several strengths, including the usage of a large sample size, the use of validated assessment instruments, and finally, utilization of a longitudinal population-based sample.

The findings also have to be interpreted in the light of some limitations. Symptoms of anxiety, depression, and NDDs in childhood, and internalizing symptoms in adolescence were assessed via parent report, which may have caused differences in response rates regarding parents who responded at age 9 and 15, and who did not. That is, a parent may not have answered because their child has a more profound NDD and/or anxiety/depression symptomatology. Alternatively, parents may have been more inclined to participate in the study and exaggerate on the questionnaires because they wanted their child to get help. Thus, results should be interpreted with caution regarding generalizability. Related to this, it was not examined which children in CATSS were treated for their childhood NDD and/or anxiety/depression symptoms, which may have theoretically masked an association in the present study. Roughly half of the parents who responded in CATSS-9/12 answered in CATSS-15, and, as reflected in the attrition analyses (see Additional file 1, Supplementary Table S1) those who responded in both waves had a significantly lower NDD score, but a much less pronounced difference was found in SCARED and SMFQ. This is in line with other studies that have reported that non-responders have a higher degree of psychopathology [53, 54]. With parent reports in general, parents may not be fully aware of their child’s anxiety/depression symptomatology, as they are often internalized [55] and displayed symptoms may occur outside of the parents’ visibility (e.g., in school or during interaction with peers). The use of self-reports may have therefore delivered different results. It has been suggested that self-reports tend to become a more reliable source of information the older the child becomes [56]. With internalizing disorders specifically, adolescent self-reports were found to predict internalizing disorders beyond information offered by parent reports [57]. Although the use of parent reports at age 15 can thus be viewed as a limitation in our study, it has been suggested that the SDQ is able to accurately capture internalizing symptoms in both self- and parent reports [58, 59]. We used different measures to capture symptoms of anxiety, depression, and NDDs in childhood, and internalizing symptoms in adolescence, which has likely influenced the ability of these measures to capture the “same” underlying construct of internalizing symptoms at the two time points. Nonetheless, it is unclear whether using the same scale and source of report across age will deliver perfect construct validity, as the meaning of any reported measure will likely change over time. For example, depression at age 9 and 15 may be conceptualized differently – certainly on a phenotypical level – and is thus unlikely captured by the same items across age. Finally, given that our outcome was internalizing symptoms in adolescence measured on a continuous scale, we very well may have picked up ‘common’ teenage anxiety, as this is a time coupled with identity shaping and worrying [60]. Hence, stricter definitions and/or categorical diagnostic criteria defined as such in the DSM-5 and ICD-11 may have yielded different results with regards to shared explained variance.

## Conclusions

In conclusion, the results of the present study suggest that adolescent internalizing symptoms are modestly affected by symptoms of childhood anxiety, depression, and NDDs, indicating that they may represent different constructs across age. Future studies should further empirically investigate differences in etiology and trajectories of childhood versus adolescent internalizing symptoms.

## Supplementary Information

**Additional file 1. **

## Abbreviations

ADHD: Attention-deficit/hyperactivity disorder
ASD: Autism spectrum disorder
A-TAC: Autism-Tics, ADHD, and other Comorbidities inventory
CATSS: Child and Adolescent Twin Study in Sweden
NDD: Neurodevelopmental disorder
SCARED: Screen for Child Anxiety Related Disorders
SDQ: Strengths and Difficulties Questionnaire
SMFQ: Short Mood and Feelings Questionnaire
