# Supplementary material for: Internalizing symptoms in adolescence are modestly affected by symptoms of anxiety, depression, and neurodevelopmental disorders in childhood
Source: BMC Psychiatry. 2022 Apr 1;22:233. doi: 10.1186/s12888-022-03875-6 (PMC8976364; doi:10.1186/s12888-022-03875-6)
Supplement: Supplementary file 1 — Additional file 1. [file 12888_2022_3875_MOESM1_ESM.docx]

**ADDITIONAL FILE 1**

| **Supplementary Table S1**. Mean scores and standard deviation of the childhood measures in individuals whose parents only responded at age 9 (CATSS-9), versus those who have responded at both assessment waves (CATSS-9 and 15). | | | |
| --- | --- | --- | --- |
|  | **CATSS-9 only** | **CATSS-9 and 15** |  |
|  | Mean (*SD*)  *n* | Mean (*SD*)  *n* | *P*-value* |
| Anxious symptoms at age 9  (SCARED) | 4.96 (6.41)  4499 | 4.72 (5.84)  4517 | 0.112 |
| Depressive symptoms at age 9  (SMFQ) | 0.96 (2.39)  5292 | 0.82 (2.03)  5137 | 0.003 |
| NDD factor | 2.38 (3.47)  5729 | 1.86 (2.84)  5708 | 0.000 |
| * Tested with Generalized Estimation Equation fit of the data. | | | |
